# Monoclonal antibody CIS43LS sets the bar for long-acting malaria protection

**DOI:** 10.1172/JCI209790

**Published:** 2026-08-17

**Authors:** David J. Sullivan

**Affiliations:** W. Harry Feinstone Department of Molecular Microbiology and Immunology, Johns Hopkins Bloomberg School of Public Health, Baltimore, Maryland, USA.

## Abstract

Monoclonal antibodies (mAbs) targeting *Plasmodium* *falciparum* epitopes aim to address gaps in malaria prevention, with potential to profoundly impact high-risk populations. In this issue of the *JCI*, Tran et al. performed pharmacokinetic and pharmacodynamic analyses on the mAb CIS43LS, which targets a unique conserved hinge region on the sporozoite protein CSP and previously demonstrated a high level of durable protection in controlled human malaria infections. Their findings establish a solid benchmark for mAb protection, demonstrating 80% protection from liver-stage invasion for 4–6 months. A clinical correlate of protection was estimated at antibody levels over 64 μg/mL. Successful protection could also be achieved from subcutaneous injections requiring lower doses. While efficacy of individual mAbs is more straightforward to demonstrate, exploring combination approaches targeting variable regions and diverse effector functions seems prudent to address the problem of evolving microbial pathogens. Combining both mAbs and long-acting malaria drugs may improve efficacy and reduce resistance.

## mAbs: the next frontier in malaria prevention?

Progress in reducing malaria worldwide has slowed in recent years, in part due to the emergence of insecticide-resistant mosquitoes and drug-resistant parasites. Children in malaria-endemic regions are at high risk for malaria morbidity and mortality, and WHO has recently issued recommendations for RTS,S and R21 vaccine regimens with 60%–75% efficacy in preventing clinical malaria in older infants and toddlers. However, these regimens demonstrate lower efficacy in other high-risk groups, such as infants and certain adult populations. There is a need for effective and safe preventative approaches to prevent malaria across high-risk populations, and long-acting mAbs targeting the infective *Plasmodium falciparum* sporozoite stage are currently in clinical development with these gaps in mind.

mAb-mediated disease prevention is currently best studied in the context of preventing viral disease. In many viral diseases like SARS-CoV-2 and respiratory syncytial virus (RSV), preventing infection requires higher levels of antibodies than reducing severe disease progression associated with hospitalization. For SARS-CoV-2, a high level of mAbs are needed to prevent infection compared with the dosing required to prevent hospitalization (1, 2). In two studies of therapeutic convalescent plasma utilizing the same donor plasma pool, convalescent plasma treatment led to a 54% reduction in severe disease hospitalization (3), whereas the same plasma treatment was not effective at prophylactically preventing SARS-CoV-2 (4). In RSV, antibody levels for infection prevention are also six to seven times higher than the level required for severe disease reduction (5, 6). For both SARS-CoV-2 and RSV infection, the spike and fusion protein targets remain the same for the purposes of infection prevention and disease reduction.

The lethal malaria *P*. *falciparum* parasite, with more than 5,000 genes, has a vast array of proteins that differ across its many phases, including the extracellular sporozoite for liver-stage invasion; the extracellular merozoites that invade erythrocytes; the intracellular infected erythrocytic asexual ring; trophozoite, schizont, and sexual gametocytes; and the extracellular gametes to ookinete to oocyst from which sporozoites develop in the mosquito (7). Sporozoites are slender elongated cells approximately 1 μm in diameter and 10 μm long with thousands of multifunctional rod-like circumsporozoite proteins (CSPs) on the surface (8, 9). Hepatocyte infection occurs within an hour of a mosquito bite, allowing only a short window for antibodies to intervene. Natural immunity to CSP does not confer clinically significant liver-stage infection prevention, even in individuals in high endemic areas who experience dozens of productive infections in a single year (10, 11). The WHO-approved malaria vaccines RTS,S and R21 target repeat regions and a T cell epitope for CSP (12); they prevent liver-stage infections but do not directly target surface antigens on merozoites or infected erythrocytes to significantly reduce severe disease. mAbs and vaccines directed to CSP are limited in their ability to prevent infection prevention and are primarily used to limit disease after infection.

## A target in CSP’s hinge region shows promise

The NIH has been leading efforts on clinical validation of long half-life mAbs to protect against passive *P*. *falciparum* infection. Characterization of monoclonal epitopes isolated from malaria-protected participants in controlled human malaria infections (CHMIs) identified a CSP hinge region epitope that is distinct from the repeat regions and T cell epitope targeted by the RTS,S and R21 vaccines (13). The three major Fc (M435L/N441A), LS (M428L/N434S), and YTE (M252Y/S254T/T256E) mutations extend serum half-life of mAbs by 2- to 4-fold by improving pH-dependent binding to the neonatal Fc receptor and promoting endosomal recycling (14). An NIH group used the M451L/N457S LS mutation to create the mAb CIS43LS, which eliminated nearly 80% of infections in a malaria endemic area as well as in CHMI, both when delivered intravenously (15, 16) and subcutaneously (17, 18). Interestingly, the COVID-19 pandemic interrupted the first evaluation of this antibody in CHMI after the mAb infusion time point; when the trial resumed in later in 2020, the antibody was found to provide long-term protection, persisting over the unplanned gap up to 90 days (19).

CIS43 (without the LS mutation) initially binds to the single-site hinge region with a < 7.5 nM binding affinity to the *P*. *falciparum* junctional peptide 21 (residues NPDPNANPNVDPN), including its hinge residues NPDP. However, affinity of the full-length IgG to recombinant protein is 7.9 nM initially, with second-step affinity measured at 42 nM (13). This stepwise increase in affinity is explained by the initial CIS43 binding, which elicits a CSP conformational change that also prevents the proteolytic CSP cleavage that is important for parasite hepatocyte entry. This finding contrasts with studies examining viral antibody interference using a viral ligand binding to a host receptor. In these studies, in vitro sporozoite hepatocyte entry was inhibited at concentrations ranging from 50 to 100 ng/mL.

## Establishing benchmarks for the CIS43LS antibody and beyond

In this issue of the *JCI*, Tran and colleagues present a well-designed study and meticulous analysis of malariometrics, host responses, and malaria genetics on the foundation of pharmacokinetics and pharmacodynamics to yield a benchmark correlate of protection for the mAb CIS43LS (20). The pharmacokinetic and pharmacodynamic analysis utilized data from doses of 5, 10, and 40 mg/kg, which translates to 25, 50, and 200 mg for a 5 kg child and 300, 600, and 2,400 mg for adults. In a larger study of 110 individuals, there were 39 breakthrough infections at the 10 mg/kg dose and 20 at the 40 mg/kg dose. The pharmacokinetic modeling parameters were used to estimate a clinical correlate of protection from the two doses at 64 μg/mL (95% CI 49–93 μg/mL). This protection estimate is 1,000 times higher than the 50 ng/mL correlate that was approximated in vitro using hepatocyte invasion inhibition (IC_62_). The pharmacokinetics align with almost 6 months of malaria protection.

Lowering to a simulated dose to 30 mg/kg, which would be achievable by a subcutaneous route of administration, afforded protection for up to four months. The authors also noted that presence or absence of concurrent malaria bloodstream parasitemia did not affect antibody levels or outcomes. The authors’ analyses also looked carefully for possible cross-reactive non-CSP malaria proteins expressed during erythrocyte stages and found that only five of the roughly 3,000 expressed *P*. *falciparum* proteins displayed minimal binding interaction. These cross-reactive proteins do not localize to the cell surface of merozoites or infected erythrocytes and were not thought to confer any clinical blood stage efficacy. Importantly, sequence data were available from all patients with breakthrough infections and indicated no new mutations in the conserved *P*. *falciparum* CIS43LS hinge epitope. Anti-drug antibodies to CIS43LS were rare, minimal, and transient; overall, these anti-drug antibodies were interpreted to be clinically nonexistent.

Estimating the antibody serum level required to prevent infections depends on the assay used. For example, using the Ghent-CEVAC ELISA, the RTS,S vaccine threshold of 121 ELISA units (EU)/mL is estimated to prevent 50% of infections (21, 22). An analysis of nine RTS,S vaccine trials with over 5,000 participants determined a protective level of 51 EU/mL (23) measured in a single laboratory (24). The ProC6C-AlOH/Matrix-M vaccine used a different assay (total IgG against full-length PfCSP) and found a 4.1 μg/mL protective level (25), but differences in assays and laboratories prohibit direct comparisons with the 121 or 51 EU/mL RTS,S values. Indeed, while the correlation of ELISA units among assays is generally good, standardizing is difficult even among anti-CSP ELISAs (26). Furthermore, comparison between vaccine-induced polyclonal antibody responses and protective levels of CIS43LS is inherently complicated by an “apples and oranges” comparison between vaccines and mAbs correlates of protection.

## Looking forward: CIS43LS and its successors in action

Malaria vaccines for infants require multiple doses to achieve efficacy by 12 months of age. The CIS43LS mAb might have its most clinically significant application in preventing a second malaria infection immediately following severe infection or as a bridge beginning at age 6 months of age, when maternal antibodies transferred at birth begin to wane. Intermittent treatment drug therapy has been successful in populations vulnerable to malaria, such as pregnant women, infants, children aged 6 months to 2 years, children under age 5, and school-age children. mAb therapy in this context might be combined with drugs to forestall drug resistance or augment its disease-lowering effects.

Additional microbe-independent mAb strategies combine a few more efficacious mAbs into a cocktail rather than relying on single-epitope targets. A blend of Fc effector function in addition to longer half-life might enhance efficacy. For instance, using the same variable region but engineering phagocytosis, complement lysis, or cellular cytotoxicity might enhance remnant malaria-infected hepatocyte clearance after infection. Tran et al.’s work provides a foundation for evaluating relative improvements in the pharmacokinetic and pharmacodynamic properties of existing and in-development mAb strategies.

## Conflict of interest

DJS is founder, board member, and stock/option owner of AliquantumRx (macrolide for antimicrobial and malaria use) and coinventor on US patent 7,270,948 (Detection of malaria parasites by laser desorption mass spectrometry), US patent 9,568,471 (Malaria diagnosis in urine), US patent 9,642,865 (New angiogenesis inhibitors), and PCT/US2015/046665 (Salts and polymorphs of cethromycin for the treatment of disease). DJS has received royalties from Binax Inc., doing business as Inverness Medical, for HRP II and aldolase plasmids for malaria diagnostics and for malaria diagnostic monoclonals to HRP II.

## Version 1 08/17/2026

Electronic publication
